# Pathological Anatomy

**Published:** 1861-03

**Authors:** John H. Packard

**Affiliations:** Philadelphia


### PATHOLOGICAL ANATOMY.

BY JOHN H. PACKARD, M.D., OF PHILADELPHIA.

I.—*Three Cases of Softening of the Pons Varolii.* By DR. J. W. OGLE. (Reported to the Pathological Society of London.)

(1) R. H., a man aged forty-five, had been for several years subject to attacks of dyspnœa; they had lately become more troublesome, and he had complained of headache and depression. One morning he said that he had pains in the occiput, and a strange feeling in his feet. The pain increased, and he was taken to St. George's Hospital; he had trembling in all his limbs, and immediately became unconscious. Volition and sensation were alike lost, and the limbs were relaxed. The pupils were at first equally dilated, but the left one afterward became much the smaller. He died an hour and a half after his admission.

"On post-mortem examination, the cranial bones and cerebral membranes were natural; the vessels of the scalp and membranes were much distended with blood; the brain itself was natural as regards its upper part. On section it was found, however, that in the pons Varolii, extending across almost the entire width of the organ, and actually bursting through the surface of the *left* side, was a large extravasated mass of blood. This occupied about the superior three-fourths of the substance of the pons, approaching so near the band of the fourth cerebral ventricle as to give way into it on very slight pressure. The inferior fourth part of the substance of the pons Varolii (the portion, that is, close to the occipital bone) was natural in appearance. This extravasated blood extended so as to touch the upper part of the medulla oblongata. It was, moreover, continued downward into the central part of the right crus cerebelli, reaching into the cerebellum. A few large vessels at the base of the brain were atheromatous, but only slightly so. Microscopical examination did not reveal evidence of any softening or alteration in the capillaries of the neighborhood."

(2) J. C., a man aged fifty, had been in St. George's Hospital for about six weeks, with pleurisy. He was discharged pretty well, but subsequently had a

"fit," and was found on the floor insensible; soon afterward he became hemiplegic, and was readmitted into the hospital, with complete loss of sensation and voluntary motion on the left side. The limbs were quite lax, the right eyebrow and corner of the mouth depressed, and articulation lost. His urine had to be drawn off daily, and became slightly albuminous; coma set in, and he died.

"On post-mortem examination, it was found that much subarachnoidean fluid existed, and the brain was what is often termed 'wet.' The cerebral hemispheres, and the fornix and central parts, were natural; the pons Varolii was, however, very much softened in the centre, the softened part being of a cream color and easily washed away. The basilar artery was very atheromatous, and was also *irregularly dilated.* All the large arteries of the brain were also atheromatous. Moreover, in one of the cerebellar arteries was a block of fibrin, tolerably firm and blood stained, completely obstructing it. The heart's walls and its valves presented nothing worthy of remark; there was a largish blood-clot in the left ventricle. The lungs were healthy; no pleural adhesions existed. The kidneys were slightly congested. Other organs natural.

"*Remarks.*—It is worthy of note that in this case, while a full plugging of a cerebellar arterial branch existed—causing, no doubt, the atrophic softening of the anterior part of the pons Varolii,—yet nothing like fibrinous fringes or deposits were found on the valves of the heart or their appendages. Of course there can be no doubt that 'such may have previously existed, and have been removed.'"

(3) W. R., a plumber, aged fifty, had had two attacks of lead colic, the first twenty years ago, the other some years later. After the second he had a fit, lost consciousness, and was very violent for twelve hours; his recovery from this was perfect, and he remained well except for occasional pains in the limbs. "On the day of admission, while on a scaffold, in the *recumbent position,* painting a roof, he was seen to roll off, and was found insensible. On admission, the surface was warm, excepting the right leg and foot, the temperature of which was much lower, and the superficial veins of which were more turgid than on the other side. There was decided lead gum-line. Suffocation was produced in attempts to swallow, and the left aperture" (ala?) "of the nose was more sunken than the right one. No movements of the alæ nasi existed during respiration. The impulse of the heart was very great, and visible pulsations existed in the right carotid and subclavian, while the left carotid could hardly be felt. No tremors existed, but frequent tetanic spasms, between which the muscles of the limbs were very rigid. There was total loss of voluntary power, and of sensibility of the skin. Much reflex action on tickling the sole of the left foot, but not so on the right side. The right pupil was dilated and oval; subsequently, the left became dilated. The tetanic spasms and muscular rigidity ceased, and the patient gradually died, the right pupil becoming more dilated than the left.

"At the post-mortem examination, a very large extravasated clot of blood was found in the substance of the right cerebral hemisphere, and also filling the third and fourth ventricles. Moreover, there was a small quantity of extravasated blood and some degree of softening in the centre of the pons Varolii, where also some of the veins were very large and dilated. The arteries of the base of the brain were very atheromatous. The left ventricle of the heart was much hypertrophied. The kidneys were atrophied and granular. The liver contained a large hydatid cyst.

"*Query.*—How far was the difference in the pulsation of the blood-vessels in this case attributable to modification of vaso-motor influence, the result of central nervous injury—or to rigidity of muscle affecting the large vessels by pressure?"—(*Lancet*, December 29, 1860.)

II.—*Tubercular Disease of the Walls of the Heart.* By DR. DA COSTA.

A colored boy, aged fourteen, had been an inmate of the Blockley Almshouse for several months. He had undergone resection of the elbow, and subsequently amputation of the same arm, for strumous disease. On the left side of his face he had a scrofulous tumor involving all the larger salivary glands, with a fistulous opening over the jaw. He was transferred from the out-wards to the Hospital, May 7th, 1860, and died three days afterward, having complained only of weakness, with a trifling cough, and some shortness of breath.

At the post-mortem examination, tuberculous deposits were found at the apex of each lung, and pleuritic adhesions on the left side. The liver was enlarged, and of the ordinary "nutmeg" appearance. The brain was soft, and its ventricles contained a good deal of fluid. Serum also existed in considerable quantity in the abdominal cavity.

But the most interesting lesion was observed in the heart. "The walls of the right ventricle possessed somewhat more than their normal thickness. In parts no muscular substance at all remained, the entire wall having been converted into a yellowish or cheesy mass lined on each side by serous membrane. In other portions there was a thin layer of muscle. The right auricle was in the same state. The left auricle and ventricle were much hypertrophied; the wall of the latter measured in parts nearly three-quarters of an inch; here and there only it contained a few deposits. There were more in the left auricle. The endocardium was smooth; but from the lining membrane of the lower portion of both ventricles sprang delicate villous growths. The valves were sound. The papillary muscles did not seem to have been involved in the disease. The pericardium was in a few spots slightly raised by the deposits under it; but the membrane itself was not roughened. It was only at the back of the right ventricle, and near the auricle, that there existed a few scattered tubercles on the surface.

"Microscopically examined, the muscular fibres on the right side were found to be very granular, their markings indistinct; some of them were covered with oil-drops. The latter were also detected in the yellowish masses, in addition to granules, and many small non-nucleated cells. No fibrous tissue was seen in the field of the instrument."

Dr. Da Costa stated that "he had met before with cases of tubercle of the pericardium, but never with tubercular formation in the walls of the organ. It was strange that the pericardium should have been so nearly healthy. In most of the cases of tubercle of the heart recorded (and they are not many) the morbid matter is formed on the external surface, and then becomes gradually imbedded in the muscular substance."

He also remarked upon the slight and recent character of the tubercular deposits in the lungs.—(*Proceedings of the Pathological Society of Philadelphia*, in the *Am. Journal of Med. Sciences*, October, 1860.)

III.—*Popliteal Aneurism; Rupture of the Aorta; Sudden Death.* By Prof. A. B. D'ALMEIDA, of Oporto.

John Oliveira, aged forty years, a blacksmith by trade, entered the infirmary of the Surgical Clinic on the 24th of February, 1859. He had scars from buboes in either groin, and other marks of a syphilitic taint. In the right popliteal space there was a tumor as large as a fist, having all the characters of an aneurism; this had been perceived for the first time five weeks previously.

Digital pressure, and the indirect pressure recommended by Broca, were alternately employed for some days; but on the second of March, after breakfast, he suddenly turned pale, and died in about five minutes.

An autopsy made the next day showed distention of the pericardium by about two pounds of blood, partly coagulated. The cavities of the heart were healthy, but its tissue was somewhat soft. An opening had occurred from the aorta into the pericardial sac, close to the origin of one of the coronary arteries; here an ulceration, involving all the coats of the aorta, was observed. The coats of all the large vessels seemed considerably softened.—(*Gazeta Medica do Porto*, October, 1860.)

(For a case very analogous to the above, see the Abstract of Path. Anatomy in the January number of this REVIEW.—P.)

IV.—*Empyema with Consecutive Pneumothorax, without Perforation of the Pleura.* By DR. KELLER.

A male child, aged ten months, had suffered for several months with diarrhœa, a hacking cough, and fever. Dr. Keller saw him on the 19th of August, 1860, and noted dullness over the whole of the left chest, with entire absence of respiratory murmur: the right side being normal. On the nineteenth of September, a clear, hollow sound was detected over the upper anterior part of the left lung; respiration had not returned. There was now less cough, but the dyspnœa was greater; the diarrhœa increased, and the child was seen in a dying condition on the twenty-fourth.

At the post-mortem examination, no communication could be made out between the pleural cavity and the air. "In opening the left side of the chest a distinct hissing from the escape of compressed air was heard. The heart was pressed toward the right side. The cavity of the left side of the chest was half empty, the other half containing about six ounces of thick, white pus. The lung was pressed against the spinal column. The pleura seemed to be very much thickened, and partly covered with a thin layer of exudation. The tissue of the lung was normal, but contained not a trace of air. The upper lobe of the right lung was pale, and emphysematous at its edges. The two lower lobes were gorged with blood and serum, particularly at their posterior portions. The smaller bronchi of the right lung contained a thick catarrhal secretion. The heart, liver, spleen, kidneys, and bowels were extremely anæmic. The stomach contained a quantity of coagulated milk and liquid food; its fundus was changed into a gelatinous mass yielding to the least touch."

Dr. Keller thinks that the disappearance of the exudation in this case must have been due to absorption, and quotes Dr. Graves's opinion that the loss of blood necessitates an absorption of air into the vessels, which air may be again secreted into any part of the body. But, as he remarks, the absorbing power of blood for gases is greater than that of water by nearly one-third; so that the more watery the blood, the less air could it take up.—(*Proceedings of the Pathological Society of Philadelphia*, in the *Am. Journal of Med. Sciences*, January, 1861.)

V.—*Congenital Deficiency of the Diaphragm; Malposition of the Viscera.* By Thomas Robinson, M.D.

Mrs. M. was delivered of a male child, apparently at full term, February 8th, 1860. The child, when born, presented a blue aspect, which soon became intense. It lived only a few minutes, and made during life ineffectual efforts to breathe, but without heaving movements of the chest—a point which was particularly observed. Percussion was dull all over the thorax; the abdomen was hard and full, and no meconium was evacuated.

A post-mortem was made forty hours after death. "The body was intensely cyanosed. Anteriorly, the thymus and heart occupied the right side of the thorax. The pericardium being opened, the heart's apex pointed to the left side; the vessels were normally arranged. On drawing the heart forward, a small lung, in an unexpanded fœtal condition, was seen occupying the space corresponding to the angles of the ribs and vertebral column of the right side. The left side from the clavicle to the half chest was occupied by the small intestines; in juxtaposition with these lay the spleen, which was in contact with the thymus gland with its right border, separated below by a rudimentary left lung, —a thin triangular-shaped body, measuring scarcely an inch in its longest direction. The lower half of the left chest cavity was filled by the left lobe of the liver, the stomach, and other parts of the intestines; the latter being to the left, the liver next, and the stomach adjacent to the pericardium and below the spleen. The diaphragm was deficient of the left tendinous part, admitting part of the left lobe of the liver and other viscera mentioned, through the space. The left crus was present, and formed a pillar, round which the œsophagus curved to rise to the stomach. The greater curvature of the stomach was superior. The right half of the diaphragm was normal. The right lobe of the liver was large, and with the kidney occupied entirely the right side of the abdomen. The left side was filled by intestine and kidney, the colon being wholly on the left side, ascending on that side of the spine into the thorax. There was no other malformation or displacement.

"Comparison with the lower vertebrata shows with tolerable clearness that this was a case of arrest of development of the diaphragm; this muscle being developed from circumference to centre, and not existing in the early life of the human fœtus, the condition permanent in birds, reptiles, and fishes. The cetaceous and amphibious mammalia have an imperfect diaphragm, approximating that of birds, which consists of bands of muscular fibres passing from the ribs to the pleura. In the porpoise there is no central tendon; resembling early life in the human fœtus and some cases of congenital deficiency. Fishes have a muscular septum between the branchial apparatus and abdomen. The true mammalia alone have a genuine diaphragm."

In connection with this very interesting case, Mr. Robinson refers to twenty-four published observations of an analogous character, and gives a tabulated statement of them. In one instance, although the diaphragm was entirely absent, life was prolonged seven years.—(*British Medical Journal*, November 3, 1860.)

VI.—*Cancer of the Œsophagus.* By MR. WOODMAN.

John G., aged sixty-two, was admitted into the London Hospital under Dr. Davies, October 9, 1860. He was much emaciated, and unable to take any solid food whatever, vomiting the greater part even of any liquid taken by the mouth; his first complaint was about three months previously, when pain and difficulty in swallowing came on. There were no signs of aneurism. A week after his admission, a little blood was found in the fluid vomited by him; and this was noticed daily for four days, when a sudden and copious discharge of bright-colored blood (about three pints) destroyed his life.

An autopsy was made, twelve hours after death. "Heart small, all its cavities contracted. On tracing the aorta to that part of the descending portion underneath the left bronchus, the portion in contact with the œsophagus was found split on its outer surface, to about a third of its circumference, and a lamina about two lines in depth, and corresponding to the split, was separable for about one-third of an inch on either side. Its margins toward the split were frayed, and the corresponding part internally had a small pin-hole aperture, surrounded by a blackish margin, easily lacerable. On removing the œsophagus at the part contiguous to the diseased portion of the aorta, a cancerous mass (scirrhus) was found, which had ulcerated through at this part, and on opening it, it was found to implicate the whole circumference of the œsophagus, admitting only a goose-quill, very dense and ragged, and about half an inch in depth. The bronchial glands were much enlarged, and one of them was occupied by a calcareous deposit, as big as a hazel-nut. There was no erosion of the trachea or bronchial tubes."—(*Medical Times and Gazette*, December 29, 1860.)

VII.—*Cancer of the Liver.* By DR. CHARLES C. LEE.

Richard Johnson, a Dane, aged forty-seven, was admitted into the Philadelphia Hospital, July 24, 1860, with enlargement of the liver. He said that his abdomen had begun to swell only five weeks previously, and that he had never had remittent fever, or any form of hepatic disease. Treatment had no effect, the enlargement steadily progressing; on the first of August he began to fail, and died on the eighteenth, having suffered no pain, and free from jaundice until the day before his death.

At the autopsy the heart was found very soft and fatty, its right side full of firm yellow fibrinous clots, entangled among the fleshy columns, and apparently of *ante-mortem* formation. About f℥ij of serum existed in the pericardium.

"Nearly three pints of limpid serous effusion were found within the peritoneum, but nothing abnormal in the stomach or intestines. The liver was enormously enlarged, completely covering the stomach, and extending deeply into the left hypochondriac region. It weighed fourteen pounds, and measured sixteen inches and a half across the under surface from right to left; the right lobe was twelve inches long and five thick. The proper hepatic structure was in great measure displaced by circumscribed deposits of medullary cancerous tissue of a yellowish-white color, marbling the surface in a beautiful manner, and rising into nodules in every direction, but so soft as to be easily compressible, and imperceptible through the abdominal walls. The microscope revealed in this structure no fibrils, but numerous caudate and multiform cells, evidently of the cancerous type, intermingled with a few hepatic cells, the former greatly preponderating in number."—(*Proceedings of the Pathological Society of Philadelphia*, in the *Am. Journal of Med. Sciences*, January, 1861.)

VIII.—*Abscess of the Liver Opening into the Right Lung.* By DR. S. W. MITCHELL.

J. S., aged thirty-seven, had been more or less ailing for two years past, with symptoms of pulmonary irritation. In March, 1860, he had an attack of acute hepatitis. In June he was surprised at a sudden increase of his previously slight cough, with a very copious purulent expectoration. Dr. Mitchell saw him on the twenty-third of July.

He was then spare and a little sallow, but not jaundiced; his strength was not much impaired, his tongue clean, his appetite and digestion good. He attributed his ailments to the foul air to which he had been exposed while in Nashville, Tenn., two years previously; there having been a large drain and some water-closets close by the printing-office in which he was engaged. Dullness on percussion was noticed over the lower lobe of the right lung in front, and over the side and back as far up as the scapular spine. At the lower angle of the scapula there were the usual signs of a cavity. Mr. S. died from exhaustion, on the twenty-ninth of August.

The autopsy was made by Dr. Kane, in presence of Drs. Meigs and Packard. Firm white clots were entangled with, and closely adherent to, the columnæ carneæ and chordæ tendineæ of both sides of the heart, the muscular fibres of which organ were much softened.

"The inferior lobe of the left lung was studded with miliary tubercles. It was much congested, and of a deep-red color. The upper lobe, though congested, was less so than the lower, and contained no tubercles. The upper lobe of the right lung contained several small cretified tubercles, but was otherwise healthy. The lower lobe was completely riddled by an anfractuous vomica. What remained of its substance was much softened and of a dirty-brown color. The pleura round this portion was much thickened; and immediately above, its two surfaces were closely adherent, thus forming a circumscribed empyema connected with the abscess of the lung.

"The liver was almost double its normal size, its left lobe extending so far into the left hypochondriac region as to press strongly against the spleen. A rough measurement, made before removing the organ from the abdomen, gave ten inches as the vertical diameter of the right lobe, nine inches as that of the left, and eleven inches as the transverse diameter of the entire viscus at its central portion. The upper left angle of the left lobe was the site of an abscess about as large as an ordinary hen's egg, which bulged outward so as to press strongly against the diaphragm, and was filled with thick, homogeneous, yellow pus. The right lobe was firmly adherent to the right wall of the abdomen and to the diaphragm, but especially to the right abdominal wall. An abscess as large as a Sicily orange occupied the upper portion of this lobe. It was filled with thick, whitish pus, and did not communicate either with the other abscesses in the liver or with the lung. A third abscess, nearly as large as a hen's egg, existed in the lower portion of the right lobe of the liver. This abscess communicated, by a large opening in its posterior wall, with the gall-bladder, which was firmly agglutinated to the liver, and was much distended with thick, greenish pus. The walls of the gall-bladder were much thickened. The cystic duct was entirely occluded by the pressure of an enlarged gland. The hepatic duct was unimpeded, as was also the common duct, which we traced to its opening in the duodenum.

"We had considerable difficulty in detecting the opening of communication between the liver and the lung, which was not, as might have been expected, above, from one of the large abscesses pressing against the concavity of the diaphragm, but by a small canalicular opening connected with the abscess in the lower portion of the right lobe, which pierced the liver low down at about the junction of its right lateral and posterior surfaces, and allowed the pus to escape. This, being circumscribed by lymph, had burrowed upward, and perforated the diaphragm at its attachment to the ribs anteriorly, thus opening into the circumscribed empyema in connection with the anterior surface of the right lung. There was considerable general peritonitis, especially in the ileocœcal region; but the stomach and intestines appeared healthy. The spleen was normal; the kidneys enlarged and pale."—(*Proceedings of the Pathological Society of Philadelphia*, in the *Amer. Journal of Med. Sciences*, January, 1861.)

### IX.—*Ulceration and Stricture of the Colon.* By DR. ALONZO CLARK.

A gentleman, sixty-six years of age, who had for some time complained of insufficient alvine evacuations, and who, in October last, suffered from painful constipation, underwent great fatigue on Christmas day, and was obliged to take to his bed. His chief symptom now was constipation, with pains in the bowels, which pains were most marked in the right iliac fossa. For several days he remained in much the same state, but then severe pain occurred, and abdominal swelling and tenderness was left behind it. No motion of the bowels could be procured, either by cathartic medicines or by enemata; it was evident that much of the swelling was due to gaseous accumulation. No vomiting took place from first to last; his pulse gradually became more and more frequent, till it rose to one hundred and thirty-six.

On the seventh day, at eight P.M., he complained of sudden pain, as if something had given way; and after this, the tympany was increased, the tenderness remaining about the same. Repeated injections at last procured copious discharges of fecal matter, but he gradually sank, and died on the 4th of January, 1861.

An autopsy was made two days afterward. The descending colon was found to be constricted at a point nearly opposite the lower extremity of the kidney, the constriction being of a dark color, very much as if black thread had been tied around the gut.

This constricted portion was carefully dissected out, and the intestine opened from above downward, and from below upward to the point of stricture; in this way it was ascertained that chronic ulceration had involved nearly the whole circumference of the bowel. "It seemed evident that this ulcer had been advancing in one part while it was cicatrizing in another, the consequence of which was a very marked contraction of the whole tube, so that the point of the little finger could barely be inserted into it. At the inferior verge of the obstruction was what appeared to be a vegetative" (?) "production, which grew directly out against the inferior border of the stricture, and in its natural position formed a sort of plug to the passage from below upward. Beyond this the colon presented nothing remarkable. At the cæcum and first portion of the ascending colon there was very great distention. Taking out the portion above and below, and tying up" (?) "the contents, we found them to be fluid in character, and, in the main, the same as the copious dejections that took place during life; but in addition to that, there were found in this sac cranberries unbroken, in I don't know what numbers. We picked out oats, with the husk on, and a great number of little flexible sticks of some sort or another, that had been taken by the mouth; the stem of an apple, etc. We afterward ascertained from the family, that he had eaten no cranberries for four weeks; they had no recollection whatever when he had eaten oatmeal gruel. But a curious circumstance noticed here was that the intestine had been ruptured upon its peritoneal coat, the surface had been rent a distance of fully five inches, and had slid upon the other tissues so as to leave a space between the rent edges equal to an inch and a half in width, and producing in the muscular coat which was thus exposed several little points of ecchymosis. At one spot in that denuded portion was a place not quite so large as a three-cent piece, covered with a grayish material, which, when scraped off, left a depression like an ulcer. There was also a corresponding appearance on the mucous surface of the intestine at the same point, so that there was left a mere film of areolar tissue, which was perforated with minute openings." Air and the fluid contents of the gut had passed through these orifices when the tube was squeezed, before it was laid open.

Very slight peritoneal inflammation existed, and less about the seat of the perforation than elsewhere. A great deal of fetid gas was contained in the serous cavity.

Dr. Sands examined the growth microscopically, and regards it as of the nature of epithelioma.—(*Proceedings of the New York Pathological Society*, in the *American Medical Times*, January 19, 1861.)

X.—*Fungus Hœmatodes of the Uterus and Ovaries in a Girl Twelve Years of Age.* By DR. CORSE.

This patient at first complained of vague abdominal pains, which did not yield to treatment. A tumor was then discovered descending far into the pelvis. The case progressed rapidly, and the abdomen became so enormously distended that tapping was performed, and three pints of a ropy, sanguineous liquid were withdrawn, with great relief. On microscopic examination, elements like those generally seen in cancer were found in this liquid.

The abdomen soon refilled; vomiting of matter like coffee-grounds came on, and she died, five or six weeks only from the first discovery of the disease.

"On post-mortem examination, the next day, a quantity of sanguineous fluid, like that obtained by the previous operation, was first evacuated; then a soft, pultaceous mass occupied the opening in the abdomen; and transverse incisions, laying open the abdomen in four flaps, showed the entire space filled with a soft cancer matter, even up between the stomach and diaphragm. The whole uterus and ovaries were in one soft mass, a part only of which would hold together to be removed. The once tolerably hard tumor had, as is usual with these growths, become quite soft with its increase in size. The greater part of the fungous growth was scooped out with a saucer, and we could then see that the walls of the abdomen were the seats of attachment for this matter; numerous tumors of all sizes, from that of a small shot to an inch or more in diameter, were found to be appended to the omentum and to the intestines and mesentery.

"The whole substance removed was placed in a common bucket, and weighed eleven pounds and a half. The fluid which escaped when the abdomen was opened was supposed to be about two pounds, and the portion remaining in the abdomen about one pound, making in all *fourteen* pounds and a half." Cancer was not known to have occurred before in the family.—(*Transactions of the College of Physicians of Philadelphia*, in the *American Journal of Medical Sciences*, January, 1861.)

